# Design and validation of *Dolosigranulum pigrum* specific PCR primers using the bacterial core genome

**DOI:** 10.1038/s41598-023-32709-y

**Published:** 2023-04-14

**Authors:** Maliha Aziz, Amber Palmer, Søren Iversen, Juan E. Salazar, Tony Pham, Kelsey Roach, Karsten Becker, Ursula Kaspar, Lance B. Price, Sharmin Baig, Marc Stegger, Paal Skytt Andersen, Cindy M. Liu

**Affiliations:** 1 Antibiotic Resistance Action Center, Department of Environmental and Occupational Health, Milken Institute School of Public Health, George Washington University, 800 22nd Street NW, Washington, DC 20052 USA; 2 Department of Bacteria, Parasites, and Fungi, Statens Serum Institut, Artillerivej 5, 2300 Copenhagen, Denmark; 3 Friedrich Loeffler-Institute of Medical Microbiology, University Medicine Greifswald, Greifswald, Germany; 4 Institute of Medical Microbiology, University Hospital Münster, Münster, Germany

**Keywords:** Molecular biology, Biomarkers, Molecular medicine, Infectious diseases, Data mining, Genome informatics, Phylogeny

## Abstract

*Dolosigranulum pigrum*—a lactic acid bacterium that is increasingly recognized as an important member of the nasal microbiome. Currently, there are limited rapid and low-cost options for confirming *D. pigrum* isolates and detecting *D. pigrum* in clinical specimens. Here we describe the design and validation of a novel PCR assay targeting *D. pigrum* that is both sensitive and specific. We designed a PCR assay targeting *mur*J, a single-copy core species gene identified through the analysis of 21 *D. pigrum* whole genome sequences. The assay achieved 100% sensitivity and 100% specificity against *D. pigrum* and diverse bacterial isolates and an overall 91.1% sensitivity and 100% specificity using nasal swabs, detecting *D. pigrum* at a threshold of 1.0 × 10^4^ *D. pigrum* 16S rRNA gene copies per swab. This assay adds a reliable and rapid *D. pigrum* detection tool to the microbiome researcher toolkit investigating the role of generalist and specialist bacteria in the nasal environment.

## Introduction

*Dolosigranulum pigrum* is a gram-positive, non-spore forming bacterium from the family *Carnobacteriaceae*^1^ commonly found in the human nasal cavity^2,3^. First described in 1993 as small, white colonies that displayed beta-hemolysis^1^, *D. pigrum* remains poorly understood and the only species of *Dolosigranulum* known to date. Epidemiologically, *D. pigrum* has been associated with the healthy state of the nasal microbiome^3,4^*.* Specifically, upper airway colonization by *D. pigrum* is negatively associated with *Staphylococcus aureus* carriage^5–9^. More recently, *D. pigrum* was found to be in higher abundance in the nasopharynx of patients with asymptomatic SARS-CoV-2 infections than patients with more severe symptoms^10^.

Rapid and cost-effective methods for the identification of *D. pigrum* are needed to facilitate future clinical and in vitro studies. Standard biochemical methods are expensive and time-consuming, as are sequencing-based methods. Matrix-assisted laser desorption/ionization-time of flight (MALDI-TOF) analysis is cost-effective, but cannot be used to detect *D. pigrum* directly from clinical samples. Using a core-genome based approach, we designed and validated a PCR-based assay that can be used to confirm *D. pigrum* isolates and detect the presence of *D. pigrum* directly from clinical samples.

## Results

### *Dolosigranulum pigrum* phylogenetic and core genome analysis

We first analyzed the genetic diversity of 21 *D. pigrum* whole genome sequences available (n = 7 from NCBI and n = 14 from in-house *D. pigrum* genomes, Table S1). We extracted 87,993 SNPs from non-recombined regions of the core genome and examined the genetic diversity based on maximum likelihood phylogeny (Figure S1). This showed multiple distinct *D. pigrum* lineages, which indicates both the non-clonal nature of *D. pigrum* and the robustness of the genome collection. We then generated and analyzed the *D. pigrum* pan-genome to identify 1291 core and 357 accessory genes. For potential assay targets, we focused on the 1291 core genes.

### *Dolosigranulum pigrum* assay design

**Figure 1:**
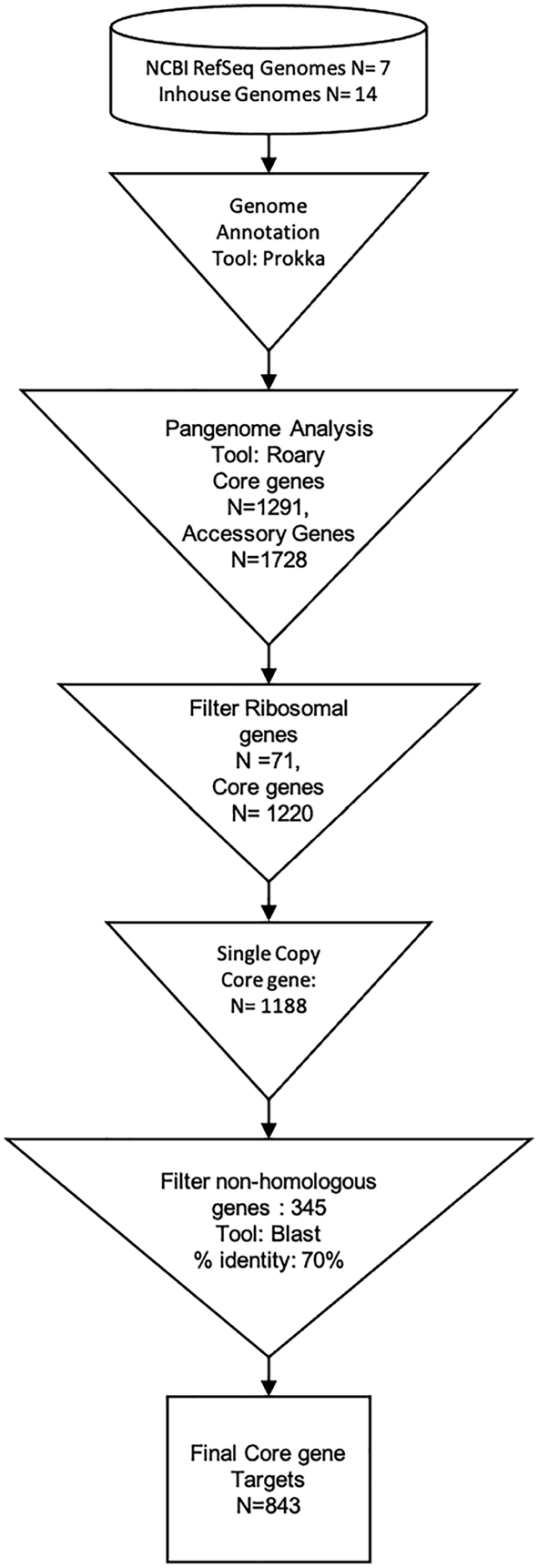
Core genome-based approach for assay design. Schematic representation of the approach taken to mine the pan-genome for assay targets. Each succeeding step in the pangenome analysis workflow illustrates how genes were filtered to finally retain a unique core genome for the organism of interest.

Assay target genes discovery was a multi-step process (Fig. 1). We removed ribosomal genes (n = 71) and genes with homologs in other genera (n = 345). Manual filtering of randomly-chosen assay target genes from the 843 single-copy core species genes was performed, requiring that the assay target gene: (a) must be present in all 21 *D. pigrum* genomes, (b) must have less than 70% similarity identity and coverage against sequences from non-*Dolosigranulum* taxa by BLAST, (c) contain forward and reverse primer sequences meeting Primer3 design criteria and that have less than 50% similarity identity and cover against sequences from non-*Dolosigranulum* taxa by BLAST.

**Figure 2:**
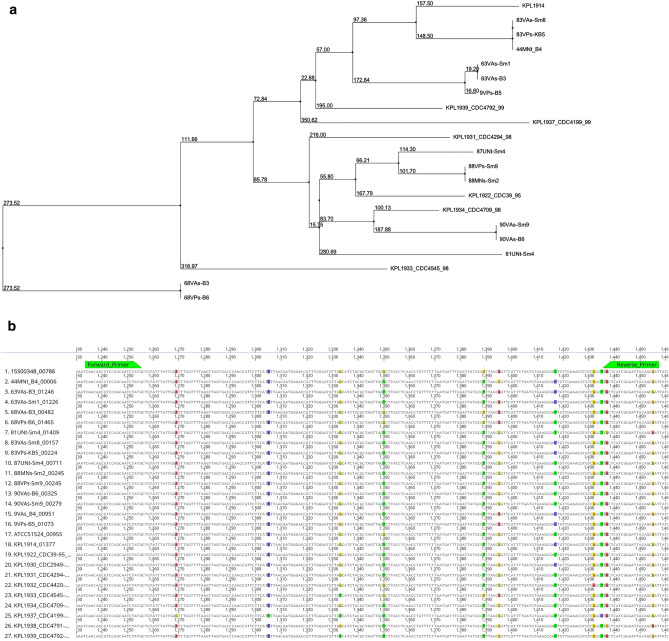
*Dolosigranulum pigrum mur*J phylogeny and sequence alignment. (**a**) Neighbor joining tree constructed using full length *mur*J gene sequences from 21 *D. pigrum* isolates using Jalview 2.11 ^37^ and ordered by branch lengths, highlighting that *mur*J is part of the conserved core genome but is also phylogenetically informative; (**b**) multiple sequence alignment of *mur*J amplicon region, where the forward primer is located at 1234–1255 bp and the reverse primer is located at 1436–1457 bp.

The first single-copy core species genes (SCSG) that met our selection criteria as a target gene candidate with conserved regions for primer design was *murJ* (Pfam ID: PF01943), a gene with a length of 1665 bp encoding a peptidoglycan lipid II flippase protein. The average uncorrected distance between the isolates for the *murJ* alignment was 35.84 bp (SD = 13.67 bp) (Fig. 2a). After iterations of primer design and in silico analysis, we identified a pair of forward and reverse PCR primers (Table 1, Supplementary Table S4a–d) targeting the *murJ* gene that produces a 223 bp PCR product. On average the amplicon varied by 2.14 bp (SD = 1.69 bp) between the isolates (Fig. 2b, Supplementary Table S3a,b, Supplementary File S1).

**Table 1:** * Dolosigranulum pigrum mur*J forward and reverse primer sequences.

Assay target	Primer	Size (bp)	Tm (annealing temp) (°C)	GC (%)	Sequence (5′–3′)
*mur*J	*mur*J_F	21	54	50	CAACAGCGTCCAGCAATCTA
	*mur*J_R	21	54	47.5	ATCGCTGTAATCCCGATRAG

### *Dolosigranulum pigrum* PCR sensitivity and specificity against clinical isolates and human nasal swabs

The *murJ* assay was highly sensitive and specific in laboratory analysis of DNA from bacterial isolates and from human nasal swabs. We first evaluated the assay using well-characterized *D. pigrum* isolates (N = 12) and against five common nasal bacterial species namely *Moraxella catarrhalis*, *Staphylococcus aureus*, *Staphylococcus epidermidis*, *Corynebacterium pseudodiphtheriticum*, *Corynebacterium propinquum*, *Corynebacterium accolens,* which showed 100% sensitivity and specificity (Figure S3).

We further evaluated the assay using DNA extracted from human nasal swabs (n = 110) characterized using 16S rRNA V3–V4 gene-based sequencing, including 54 samples that were positive for *D. pigrum* and 56 samples that were negative for *D. pigrum*. This showed that the *mur*J assay was not able to detect *D. pigrum* in samples (n = 9) with fewer than ten *D. pigrum* 16S rRNA gene copies per uL of swab eluent, or 1.0 × 10^4^ *D. pigrum* 16S rRNA gene copies per swab. However, among the 45 *D. pigrum*-positive samples with more than 1.0 × 10^4^ *D. pigrum* 16S rRNA gene copies per swab, the *mur*J PCR assay was able to detect *D. pigrum* in 41 (91%) samples (Table 2, Figs. S4, S5). There were no false positives in the 56 *D. pigrum*-negative samples.

**Table 2:** Detection of *D. pigrum* in nasal samples by PCR in relation to *D. pigrum* absolute abundance.

*Dolosigranulum pigrum* absolute abundance (16S rRNA gene copies/swab)	Positive	Negative
< 1 × 10^4^	0	58
1 × 10^4^–< 1 × 10^5^	0	7
1 × 10^5^–< 1 × 10^6^	17	3
1 × 10^6^ or greater	24	1

## Discussion

By identifying potential assay targets using the *D. pigrum* core genome, we designed a novel PCR assay that is both sensitive and specific for *D. pigrum*. In contrast to other commonly used methods for species confirmation, such as biochemical testing, DNA sequencing, or MALDI-TOF, PCR-based assays are rapid and cost-effective and do not require expensive equipment. This method provides a simpler option for *D. pigrum* detection and avoids the restriction digestion and analysis challenges of T-RFLP^11^ that has been used previously for detecting microbial communities in anterior nares^12^. We demonstrated the utility of the core genome mining techniques to develop species confirmation assays. The resultant *mur*J assay was able to identify *D. pigrum* and diverse bacterial isolates with a 100% sensitivity and specificity. Our assay was also highly sensitive and specific for detecting *D. pigrum* in clinical samples.

*Dolosigranulum pigrum* is gaining interest as a member of the upper respiratory tract microbial community that is potentially beneficial for the host^5,6,8,13–19^. Efforts are being made to better understand its metabolic models and defense mechanisms^20^. There is a critical need to screen samples to detect the presence of *D. pigrum* or to verify the identity of the organism isolated through culture-based methods. Our single step gel-based PCR method for the species verification of *D. pigrum* in clinical samples as well as pure isolates provides a useful tool for epidemiological and clinical studies.

## Methods

### *Dolosigranulum pigrum* core genome analysis

We curated a local *D. pigrum* genome database by downloading publicly available genomes from NCBI RefSeq and adding in-house sequenced and assembled *D. pigrum* genomes (Table S1). DNA from the inhouse *D. pigrum* isolates was extracted using a DNeasy Blood and Tissue kit (Qiagen) or MagNA Pure LC DNA Isolation Kit (Roche) and libraries were generated with a Nextera XT DNA Library kit (Illumina) according to manufacturer’s instructions for paired-end sequencing on an Illumina NextSeq 500 (Illumina, Inc., San Diego, CA) with a read length of 150 bp. We assembled Illumina short read sequences from inhouse *D. pigrum* isolates into contigs using the SPADES assembler (v.3.5)^21^. Quality of the assembly was assessed using metrics generated by QUAST (v.2.3)^22^ and all genomes were annotated with Prokka (v. 1.13)^23^. To maximize assay sensitivity for *D. pigrum* detection we focused on the core genome. The GFF files from the Prokka annotation step were used as input for the pan-genome analysis with Roary (v.3.12.0)^24^ [blastp v.2.9.0 identity = 90%, gene presence in isolates to be core = 99%]. We generated a maximum likelihood tree from core genome SNPs to assess relatedness of the *D. pigrum* isolates using previously described methods^25,26^. Briefly, Illumina short reads from inhouse *D. pigrum* isolates were mapped to the chromosome of the published *D. pigrum* reference genome (strain 83VPs-KB5; GenBank accession no. CP041626.1) using the NASP pipeline that uses BWA-MEM (v.0.7.12)^27^ to align and GATK (v.3.5)^28^ to call SNPs. Publicly available genomes downloaded from NCBI RefSeq were aligned to the reference using MUMMER and SNPs were identified. The resultant SNP matrix was processed with Gubbins^29^ to remove recombinant regions. A Phylogenetic tree was constructed from the core SNPs in PhyML with Smart Model selection (v.3.0)^30^. The maximum likelihood phylogeny was visualized alongside the pangenome using PHANDANGO^31^ (Figure S2). Uniprot IDs of the core genes wherever available, were extracted from the GFF files using an inhouse script and were used to retrieve Gene Ontology terms from UniProt database^32^ (Table S2). The GO terms were analyzed and summarized using GAOTools^33^.

### *Dolosigranulum pigrum* assay target identification

The core genome was filtered and only SCSG were retained. An in-silico search for homology against non-*D. pigrum* species was performed using blastn v.2.9.0^34^ using a local copy of the NT database (updated: 2019-03-31). Gene targets with 70% similarity to non-*D. pigrum* species were removed. A final set of homologous single-copy core genes was used as the candidate pool for targets to design *D. pigrum* specific assay.

### *Dolosigranulum pigrum* assay design

We used Primer3^35^ with default settings to identify candidate forward and reverse primers which were first compared to the *D. pigrum* gene alignment file then checked for similarity against other nasal bacteria, including *Staphylococcus aureus, Staphylococcus epidermidis, Corynebacterium* spp.*, Cutibacterium* spp., *Moraxella* spp., *Escherichia coli, Klebsiella* spp.*, Citrobacter* spp., *Proteus* spp., and *Alloiococcus* spp. Primers were excluded if 5 or more matching bases were found at the 3′-end of the primer.

### *Dolosigranulum pigrum* assay validation

To assess the sensitivity of our primers, we tested the *mur*J assay against 12 *D. pigrum* isolates. These isolates had been previously verified to be *D. pigrum* by MALDI-TOF and their genomes were sequenced using Illumina HiSeq system (Illumina, San Diego, CA). Furthermore, we screened *mur*J primers against 110 clinical samples characterized by 16S rRNA gene-based sequencing as described previously^36^. A non-*D. pigrum* control collection that included *Moraxella catarrhalis*, *Staphylococcus aureus*, *Staphylococcus epidermidis*, *Corynebacterium pseudodiphtheriticum*, *Corynebacterium propinquum*, *Corynebacterium accolens* species was used to evaluate specificity of our primers.

### Human subject research

Ethical approval for this study was granted by the George Washington University Institutional Review Board and The Office of Human Research. The study and its protocols were implemented according to the approved guidelines outlined in the Declaration of Helsinki. Informed consent was obtained from all participants prior to enrollment in the study.

### Human nasal swab collections

The first study included 16 healthy community-dwelling adults in Washington, DC (IRB#: NCR191444) were included. At enrollment, nasal specimens were self-collected by participants under staff guidance using Puritan HydraFlock swabs (Puritan Medical Products, Guilford, ME) with staff instructions. Samples were placed immediately into Amies transport media and stored at 4 °C until processing. Samples were processed within 4 h then transferred in 100 μL aliquots into labeled 2 mL cryovials and stored at − 80 °C. The second study included 94 healthy community-dwelling adults in Copenhagen, Denmark (IRB # 041631), which were collected by study personnel and collected into DNA/RNA shield (Zymo R1100-250) and stored at − 80 °C until processing.

### DNA isolation and purification

DNA from human nasal swabs were extracted using MagMax DNA Ultra 2.0 Kit with enzyme and chemical lysis as previously described ^5^. DNA from bacterial isolates were extracted through heat soak (*D. pigrum*, *S. aureus,* and *S. epidermidis*) or using the DNeasy Blood & Tissue Kit (Qiagen, Valencia, CA) (C. *propinquum* and *C. pseudodiphtheriticum)* according to manufacturer instructions.

### murJ PCR amplification

Each *mur*J PCR was performed in a 20 μL reaction volume containing 1 μL of template DNA added to 19 μL of PCR reaction mix containing 0.4 μM of forward (5′-CAACAGCGTCCAGCAATCTA-3′) and reverse (5′-ATCGCTGTAATCCCGATGAG-3′) primer, 1× Phusion High-Fidelity PCR Master Mix (ThermoFisher), and molecular-grade water. Amplification was performed on a C1000 Touch Thermocycler (Bio-Rad, Hercules, CA) using the following conditions: 98 °C for 30 s for denaturing, 54 °C for 30 s for annealing, and 72 °C for 1 min for extension × 35 cycles. Amplified DNA was run on a 2% agarose E-gel (ThermoFisher) to assess amplification of *D. pigrum* DNA. Gels were imaged using a ChemiDoc-It2 (Analytik Jena US, Upland, CA). Presence of a visible band at the 223 bp size indicated successful amplification.

## Supplementary Information

Supplementary Information.

## Data Availability

Raw Reads generated from the whole genome sequencing performed for this study were deposited at NCBI SRA (Accession ID: PRJNA770953).
