# Supplementary material for: Design and validation of Dolosigranulum pigrum specific PCR primers using the bacterial core genome
Source: Sci Rep. 2023 Apr 14;13:6110. doi: 10.1038/s41598-023-32709-y (PMC10103046; doi:10.1038/s41598-023-32709-y)
Supplement: Supplementary file 1 — Supplementary Information. [file 41598_2023_32709_MOESM1_ESM.pdf]

Design and validation of *Dolosigranulum pigrum* specific PCR primers using the bacterial core genome

Maliha Aziz, Amber Palmer, Søren Iversen, Juan E. Salazar, Tony Pham, Kelsey Roach, Karsten Becker, Ursula Kaspar, Lance B. Price, Sharmin Baig, Marc Stegger, Paal Skytt Andersen, Cindy M. Liu

Supplementary Results

Strain	Source	Collection year	Host	Country	Total contigs	Total bases	Total genes	NCBI Accession
KPL1914	Nasal swab	2010	Human	USA	76	1726398	1692	GCA_003263915.2
KPL1931_CDC4294-98	Blood	1998	Human	USA	82	2014679	1999	GCF_003264085.1
KPL1937_CDC4199-99	Blood	1999	Human	USA	65	1976602	1884	GCF_003264005.1
KPL1933_CDC4545-98	Nasopharyngeal	1998	Human	USA	19	1861299	1787	GCF_003264045.1
KPL1939_CDC4792-99	Nasopharyngeal	1999	Human	USA	47	1893917	1822	GCF_003263965.1
KPL1934_CDC4709-98	Eye	1998	Human	USA	82	1912682	1805	GCA_003264015.2
KPL1922_CDC39-95	Sinus	1995	Human	USA	75	1859258	1794	GCF_003264145.1
87UNt-Sm4	Nose	2013	Human	Germany	65	1954981	1874	SRR19918654
63VAs-B3	Nose	2012	Human	Germany	51	1964942	1862	SRR19918661
63VAs-Sm1	Nose	2012	Human	Germany	50	1969732	1867	SRR19918660
9VPs-B5	Nose	2011	Human	Germany	55	1903686	1819	SRR19918653
83VAs-Sm8	Nose	2012	Human	Germany	29	1918043	1893	SRR19918656
83VPs-KB5	Nose	2012	Human	Germany	39	1917955	1891	CP041626.1
44MNt_B4	Nose	2012	Human	Germany	48	1891984	1890	SRR19918652
88MNs-Sm2	Nose	2013	Human	Germany	20	1910927	1933	SRR19918655
88VPs-Sm9	Nose	2013	Human	Germany	22	1862240	1849	SRR19918663
90VAs-B6	Nose	2013	Human	Germany	44	1858197	1813	SRR19918662
90VAs-Sm9	Nose	2013	Human	Germany	38	1863257	1831	SRR19918651
68VAs-B3	Nose	2012	Human	Germany	20	1954390	1886	SRR19918659
68VPs-B6	Nose	2012	Human	Germany	50	1958590	1896	SRR19918658
81UNt-Sm4	Nose	2012	Human	Germany	1	1876539	1793	SRR19918657

Table S1. List of whole genome sequences of *D. pigrum* isolates analyzed in this study.

GO Category	Go Terms	GO ID	Count
BP	Cellular process	GO:0009987	375
BP	Metabolic process	GO:0008152	338
BP	Localization	GO:0051179	63
BP	Biological regulation	GO:0065007	49
BP	Response to stimulus	GO:0050896	40
BP	Biological process involved in interspecies interaction between organisms	GO:0044419	5
BP	Developmental process	GO:0032502	4
BP	Detoxification	GO:0098754	3
BP	Reproductive process	GO:0022414	1
BP	Biological adhesion	GO:0022610	1
BP	Signaling	GO:0023052	1
BP	Multi-organism process	GO:0051704	1
BP	Immune system process	GO:0002376	1
CC	Protein-containing complex	GO:0032991	45
CC	Cellular anatomical entity	GO:0110165	27
MF	Catalytic activity	GO:0003824	533
MF	Binding	GO:0005488	99
MF	Transporter activity	GO:0005215	87
MF	ATPase	GO:0016887	34
MF	Antioxidant activity	GO:0016209	5
MF	Transcription regulator activity	GO:0140110	5
MF	Translation regulator activity	GO:0045182	3
MF	Molecular carrier activity	GO:0140104	2
MF	Structural molecule activity	GO:0005198	1
MF	Molecular transducer activity	GO:0060089	1
MF	Small molecule sensor activity	GO:0140299	1
MF	Enzyme activator activity	GO:0008047	1
MF	Kinase regulator activity	GO:0019207	1
MF	Molecular function regulator	GO:0098772	1
MF	Molecular adaptor activity	GO:0060090	1

Table S2. Summary of Gene Ontology terms and frequency in the core genome of *D. pigrum* isolates.

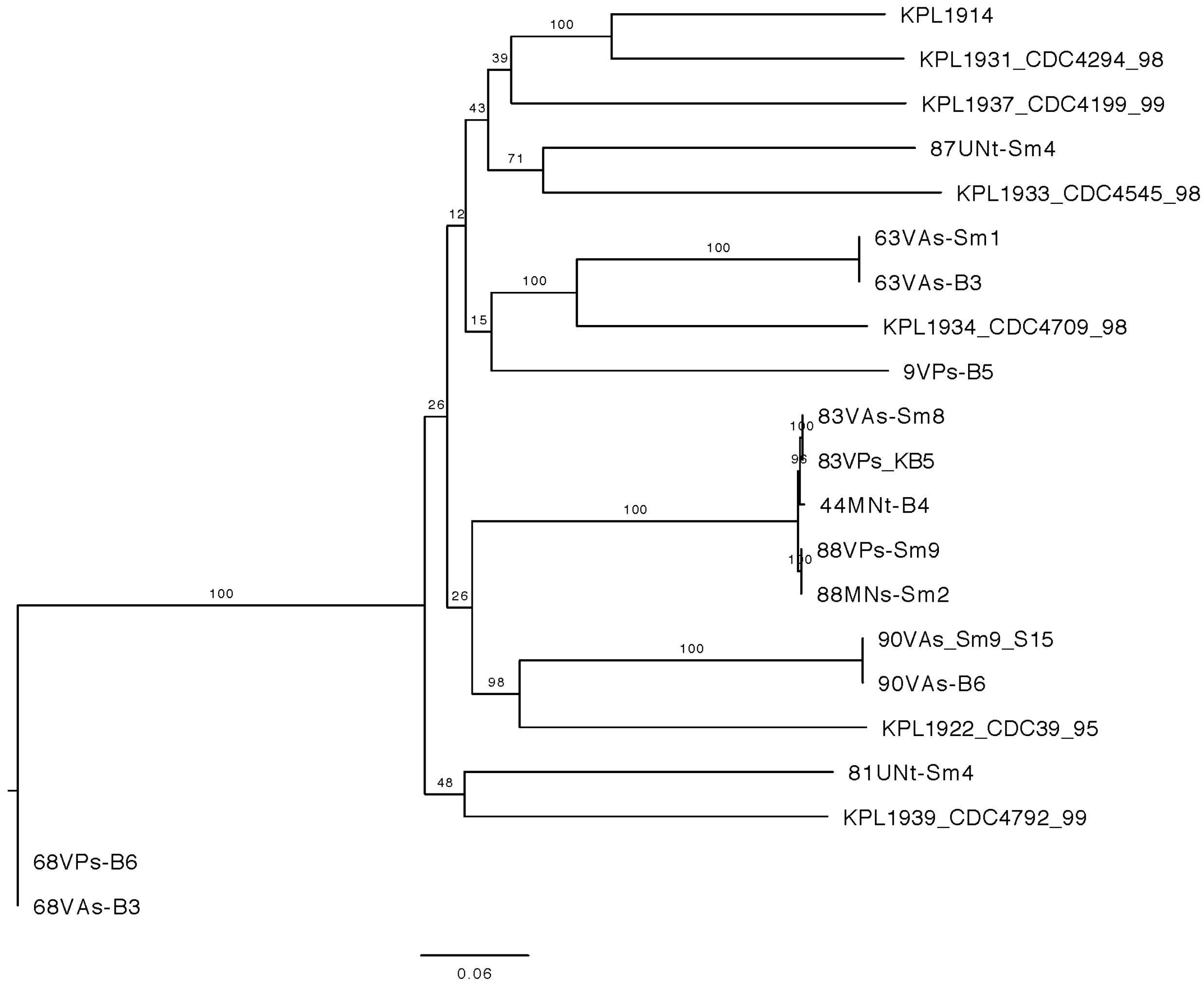

Figure S1. ***D. pigrum*** **core genome-based phylogeny.** Unrooted maximum likelihood phylogeny of 21 *D. pigrum* isolates. The tree was constructed from genome-wide core SNPs using NASP, Gubbins and PhyML. Branches show the bootstrap values.

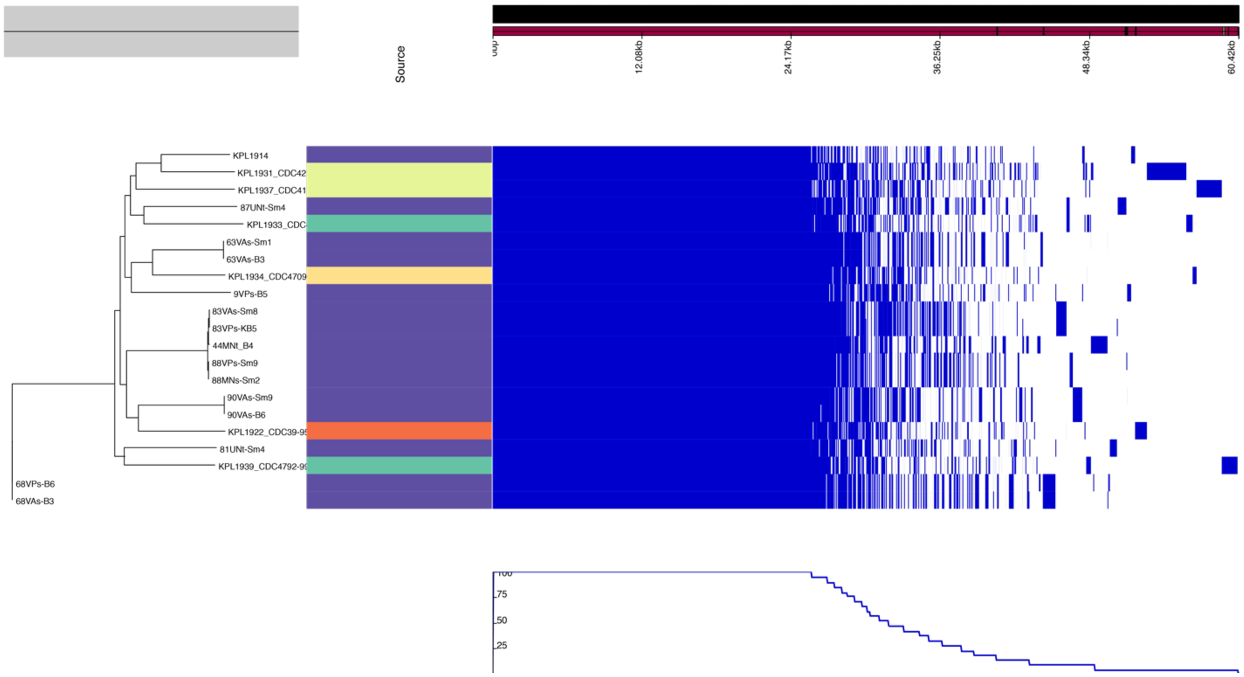

Figure S2. *D. pigrum* pan-genome. Pangenome of 21 *D. pigrum* genomes displayed with the maximum likelihood tree on the left using PHANDANGO.

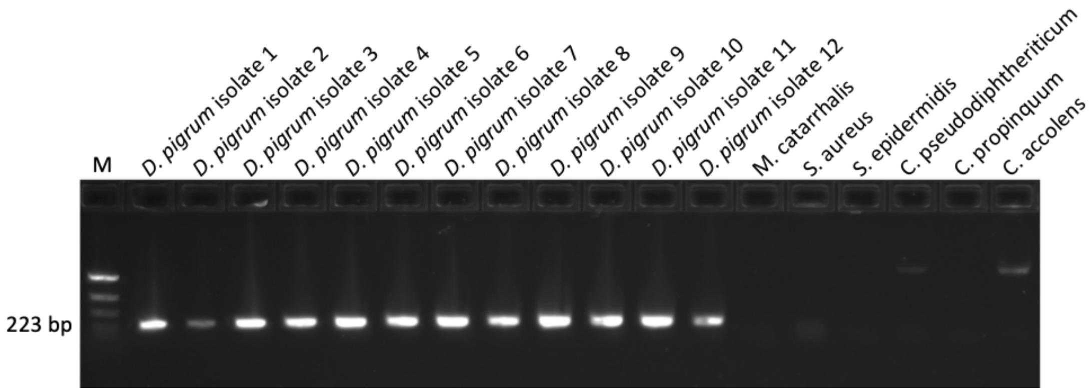

Figure S3. Specificity E-gel. Result from screening of murJ primers against a panel of *D. pigrum* and non *D. pigrum* isolate DNA.

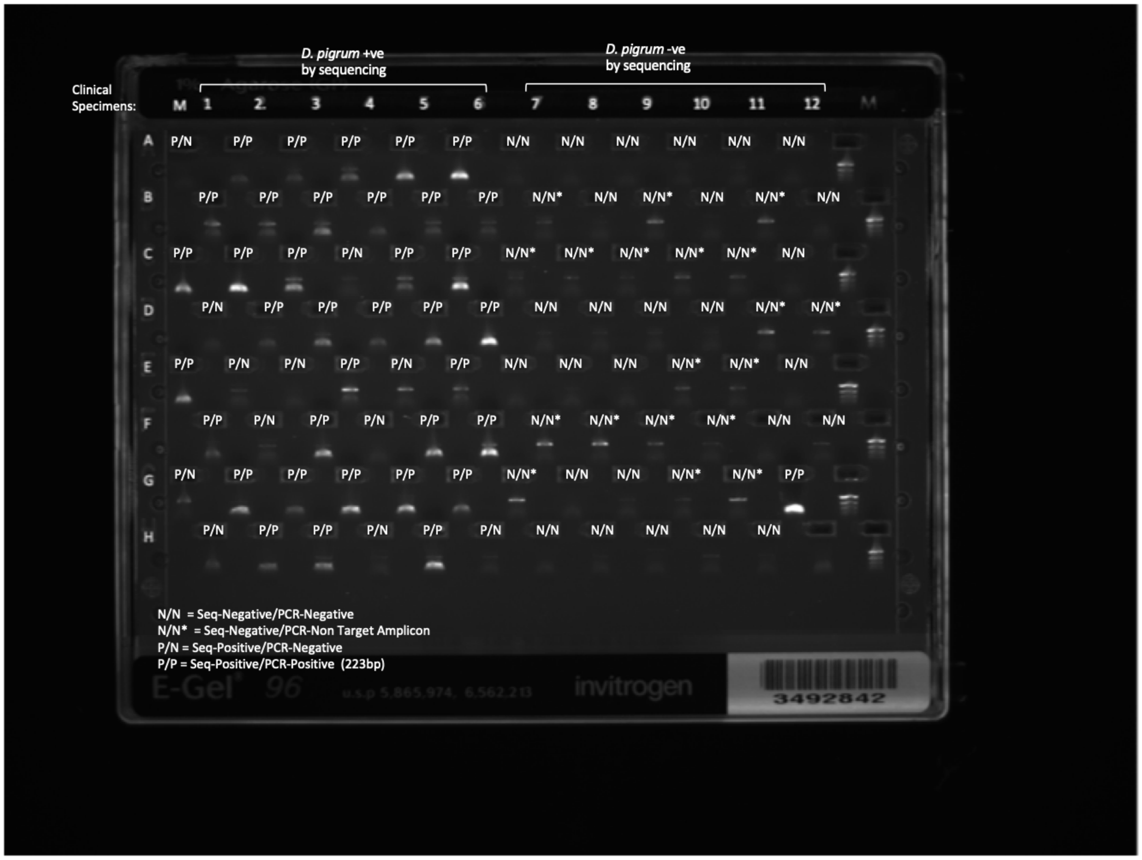

Figure S4. E-gel image for sensitivity screening. E-gel result from screening for murJ PCR amplified DNA from 94 clinical specimens collected in a study conducted in Copenhagen, Denmark. The clinical specimens were selected based on the presence or absence of *D. pigrum* in 16SrRNA sequencing data. G12 is a pure *D. pigrum* isolate and H12 is an empty well.

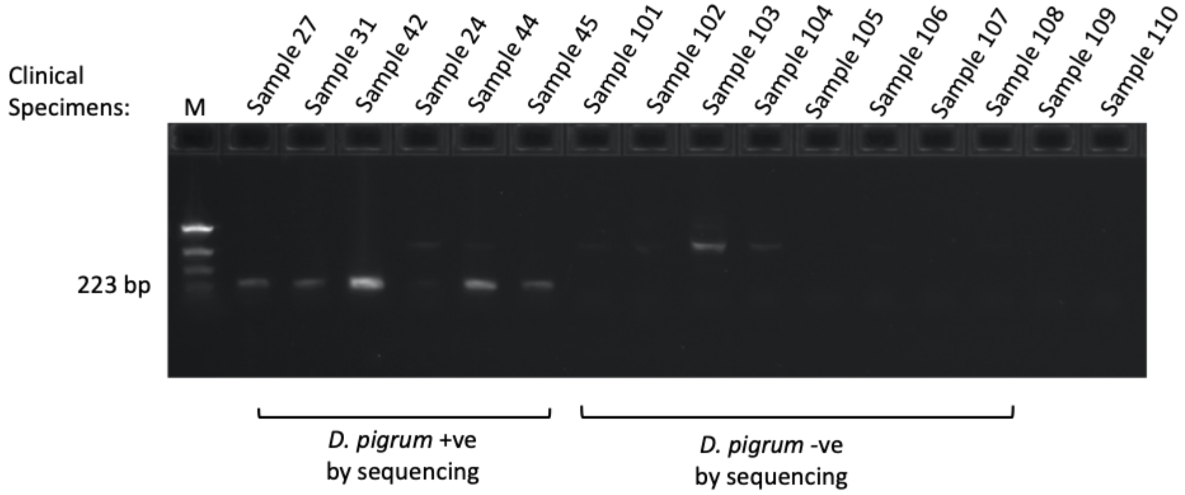

Figure S5. E-gel image for sensitivity screening. E-gel result from screening for murJ PCR amplified DNA from 94 clinical specimens collected in a study conducted in Washington, DC. The clinical specimens were selected based on the presence or absence of *D. pigrum* in 16SrRNA sequencing data
